# Incorporating Pathway Information into Feature Selection towards Better Performed Gene Signatures

**DOI:** 10.1155/2019/2497509

**Published:** 2019-04-03

**Authors:** Suyan Tian, Chi Wang, Bing Wang

**Affiliations:** ^1^Division of Clinical Research, The First Hospital of Jilin University, 71 Xinmin Street, Changchun, Jilin 130021, China; ^2^Department of Biostatistics, Markey Cancer Center, The University of Kentucky, 800 Rose St., Lexington, KY 40536, USA; ^3^School of Life Science, Jilin University, 2699 Qianjin Street, Changchun, Jilin 130012, China

## Abstract

To analyze gene expression data with sophisticated grouping structures and to extract hidden patterns from such data, feature selection is of critical importance. It is well known that genes do not function in isolation but rather work together within various metabolic, regulatory, and signaling pathways. If the biological knowledge contained within these pathways is taken into account, the resulting method is a pathway-based algorithm. Studies have demonstrated that a pathway-based method usually outperforms its gene-based counterpart in which no biological knowledge is considered. In this article, a pathway-based feature selection is firstly divided into three major categories, namely, pathway-level selection, bilevel selection, and pathway-guided gene selection. With bilevel selection methods being regarded as a special case of pathway-guided gene selection process, we discuss pathway-guided gene selection methods in detail and the importance of penalization in such methods. Last, we point out the potential utilizations of pathway-guided gene selection in one active research avenue, namely, to analyze longitudinal gene expression data. We believe this article provides valuable insights for computational biologists and biostatisticians so that they can make biology more computable.

## 1. Introduction

Data obtained from the high-throughput technologies such as microarrays or RNA-sequencing (RNA-seq) is a recurring theme in many fields such as computational biology and bioinformatics. Given these advanced technologies are expensive, the number of observations/subjects is usually small, i.e., on the scales of several to hundreds. Another special characteristic of the high-throughput technologies is that they can measure thousands of variables/features simultaneously. As far as the statistical modeling is considered, a classic regression model becomes nonidentifiable when all measured variables are used as predictors for such a data set; let alone one may also be interested in exploring the nonlinear association at higher orders or the interactions among these variables. To deal with the data in which the number of variables is extremely larger than the number of samples, the implementation of a feature selection process that identifies a subset of genes with the optimal predictive performance [1] is in demand.

Feature selection has outstanding merits. Especially, the resulting subset of genes speeds up the learning process, improves predictive accuracy, and leads to a better biological implication. The classic feature selection, we call it “gene-based feature selection” to avoid ambiguity in this article, is stratified into three subtypes, say, filter, embedded, and wrapper methods [1, 2]. These three categories have their own unique characteristics. For instance, a filter method usually screens individual features one by one according to their relevancy level with the outcome of interest [1]. The feature selection of an embedded method is usually realized by using a penalized regression model such as the Least Absolute Shrinkage and Selection Operator (LASSO) model [3]. Such a method can simultaneously select relevant features and estimate those coefficients (the effect size of those features) in the final model; in addition to that it consumes less computing time than a wrapper method.

Nevertheless, the gene subset/list selected by a gene-based feature selection algorithm has several drawbacks. First, the predictive performance on new independent samples is unsatisfactory; the overfitting phenomenon is always apparent. Second, the gene lists trained from different data sets barely overlap. Reproducibility or stability of the final models (with different data, the same method gives different gene lists with few or no overlaps) is low, leading to a generalization of the resulting gene list impossible. Last, most of these methods use the difference of gene expression level between different phenotypes as a critical criterion to select the genes associated with the outcome. However, differentially expressed genes (DEGs) are not necessarily to be true driver genes. Ignoring biological information may result in a meaningless biological implication for the resultant gene list.

Furthermore, it is well known that genes do not function in isolation but rather work together within various metabolic, regulatory, and signaling pathways. The interdependencies among genes are often represented as a collection of pathways/gene sets in which potential coregulated or coexpressed genes are grouped together. In this review, the terms “pathway”, “network”, and “gene set” have same implication/meaning and are exchangeable to one another.

The biological information contained within pathways can be utilized to impose additional constraints on the prediction tasks, forcing training methods to select more scientifically meaningful genes rather than those statistically significant genes (such as genes more differentially expressed between two phenotypes). A feature selection process that incorporates pathway knowledge by one means or another is referred to as pathway-based feature selection herein, which has currently grown into a hot topic in computational biology and bioinformatics.

So far, to the best of our knowledge, no survey on pathway-based feature selection methods in the literature has been given yet. The objective of this article is to provide a selective review on such methods.

## 2. Pathway-Based Feature Selection Methods

**Figure 1:**
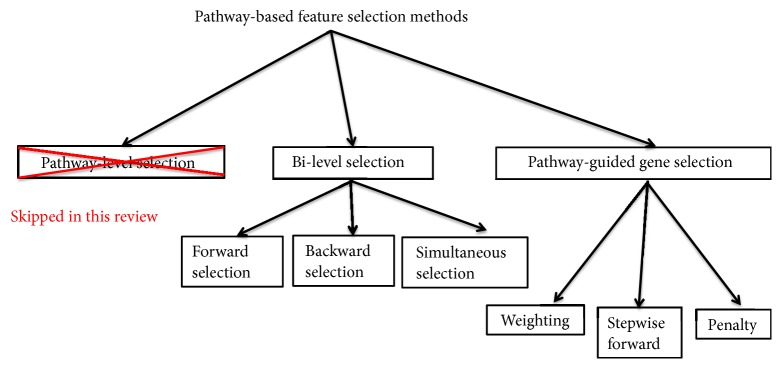
Major ramifications of pathway-based feature selection methods.

Based on to what a feature refers, pathway-based feature selection methods may be classified into three categories; see Figure 1. The first category contains pathway analysis methods such as [4, 5] in which a feature corresponds to a pathway, with the objectives of selecting the whole pathways associated with the phenotypes of interest. Since the methods in this category have been reviewed by many researchers previously and several well-known algorithms have been compared exclusively using simulations and real-world data [6–15] and our attention is mainly focused on the selection of individual genes related to the phenotypes of interest, we skip this topic in the article.

The second category considers a bilevel selection process, which identifies not only relevant pathways but also important genes that contribute critically to the significance of identified relevant pathways. The bilevel selection methods can be further divided into three major categories, forward selection, backward selection, and simultaneous selection [16]. In a forward selection process such as [10], the selection of relevant gene sets is carried out firstly and then followed by the selection of relevant individual genes. In contrast, the selection steps in a backward selection process such as [17] take the reversed order. Last, a simultaneous selection process such as [18] performs the selection of significant gene sets and the selection of important genes at the same time, as its name implies. The simultaneous selections of gene sets and genes are usually accomplished with the aids of a penalized model where a penalty term imposing some restrictions on the *β* coefficients that represent the association magnitude with the outcome is added to the objective function.

In the last category, a feature corresponds to an individual gene. The methods of this type incorporate the pathway knowledge as a priori to facilitate the selection of relevant genes, aiming to improve the resulting gene list's predictive ability and/or reproducibility. Although we had intended to reserve the syntax of “pathway-based feature selection” for this specific subfield, we frame a specific term “a pathway-guided gene selection” for it instead to avoid confusions. Given our attention is focused on the methods capable of selecting important individual genes [16], the bilevel selection algorithms, e.g., [10, 19], may be loosely classified into the pathway-guided gene selection category.

## 3. Pathway-Guided Gene Selection Methods

### 3.1. Three Major Categories

In our previous study [20], we stratified a pathway-guided gene selection method into three classes on the basis of which piece of pathway information was incorporated and how such information was incorporated, namely, weighting, stepwise forward, and penalty. In the following subsections, a detailed description of and discussion on these three categories are given.

### 3.2. Stepwise Forward

The stepwise forward methods usually rank all genes according to a specific discriminative score. Then the methods start from the most significant gene and evaluate the performance of the resulting gene subset based on some predetermined metric. The step iterates until no further gain upon this performance statistic can be obtained. A bilevel selection method, the significance analysis of microarray gene set reduction (SAMGSR) algorithm [10], can be put into this category. This method consists of two steps. Its first step is essentially an extension of the significance analysis of microarray (SAM) method [21] to all genes inside a gene set, and a new statistic called SAMGS [4] which is the square sum of SAM statistics for all genes inside a specific gene set is generated. The significance level of a gene set is determined using permutation tests. Obviously, this step carries out the selection of significant pathways firstly so that the SAMGSR method belongs to the forward bilevel selection category. In the second step, a subset of important genes is extracted from each significant pathway identified by the first step on the basis on the magnitudes of individual genes' SAM statistics. The realization of this extraction is by the means of stepwise forward. Specifically, the genes inside each significant pathway are ordered decreasingly based on the magnitude of their SAM statistics. Then the reduction step gradually partitions the entire gene set into two subsets: the reduced subset that includes the first k genes and the residual subset including the remaining genes for k = 1,…, |j|, where |j| is the size of gene set j. At each partition, the significance level of the reduced subset is evaluated using the p-value of SAMGS statistic for its corresponding residual subset. The iteration stops until this p-value is larger than a predetermined threshold for the first time.

Another typical example of a stepwise forward method is the algorithm proposed by Chuang et al. [22]. This method starts from a seed gene and identifies a gene list by gradually adding the neighboring gene that provides the highest mutual information between the average of expression values for all included genes and the outcome. In this example, network topology information that records how genes are connected instead of the grouping membership information is taken into consideration.

Two big drawbacks of a stepwise forward method are as follows: (1) the methods may fail to identify those ‘driving' genes with subtle changes because the inclusion of a gene depends largely on its expression values or expression differences among different phenotypes; (2) the selection process of important genes is usually separated from the final model construction.

### 3.3. Weighting

The weighting methods construct a pathway knowledge-based weight that reflects how important a gene is inside the gene-to-gene interaction network for each gene and then balance between the weight and its gene expression values to determine the significance level of the specific gene. For example, the reweighted recursive feature elimination (RRFE) method [23] uses the GeneRank algorithm [24] to alter the ranking criterion of the support vector machine recursive feature elimination (SVM-RFE) algorithm, and then identifies a subset with the best discriminative power. More specifically, the resulting GeneRanks are used as weights and combined with the coefficients of SVM to increase the chance of a gene with more directly connected neighbors being selected.

In the RRFE method, the weights are combined with the statistics (i.e., the coefficients in a SVM model). An alternative strategy of weighting is to combine the weights directly with gene expression values to generate weighted gene expression values and then implement a gene-based feature selection method such as LASSO to identify relevant genes. An example of this category is [25], in which the weighted expression profiles were used to classify two major subtypes of non-small-cell lung cancer. Overall, the weighting methods are the least implemented in the literature, compared to the methods in other two categories. This may be due to that the constructed weights are subject to biases and errors, which might lead to inferiority of the resulting gene lists.

### 3.4. Penalty

**Figure 2:**
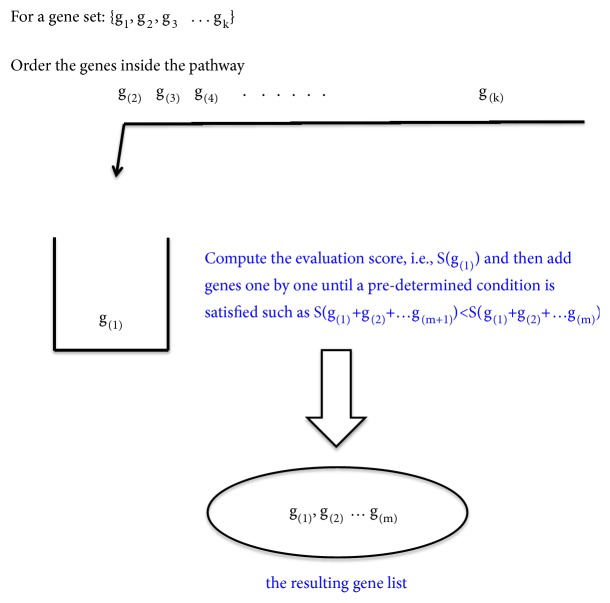
Graphical illustration of the stepwise forward methods.

**Figure 3:**
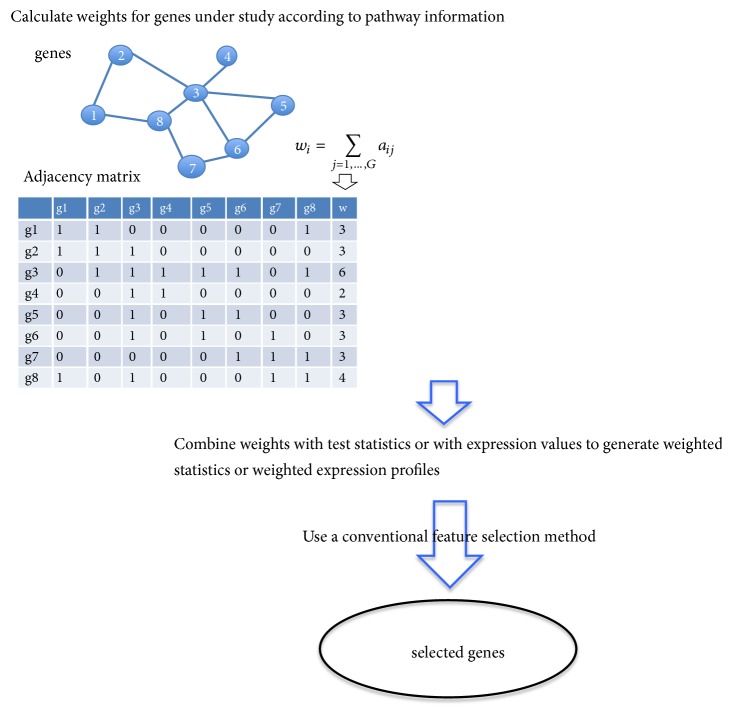
Graphical illustration of the weighting methods.

**Figure 4:**
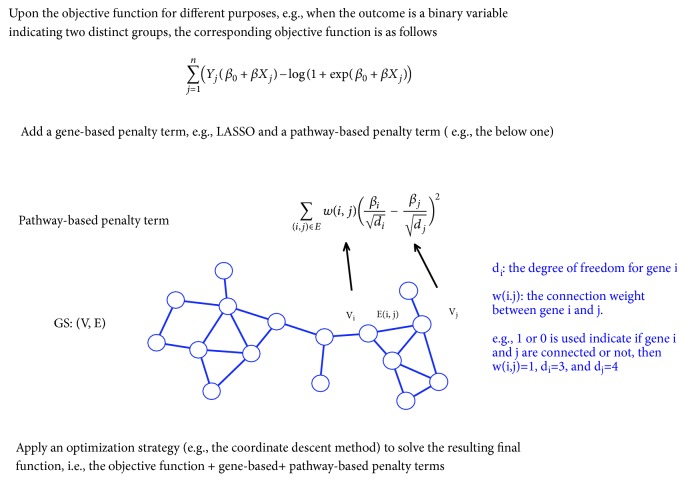
Graphical illustration of the penalty methods.

In a penalty model, an extra penalty term that records pathway information is combined with an objective function such as the log likelihood function to generate the final objective function. The identification of relevant genes is realized by the means of finding the best subset of genes that optimize this function. To name several penalty methods, Zhu et al. [26] combined the network-constrained penalty term given by [27] with a SVM model and proposed the network-based SVM method to discriminate two different phenotypes. Similarly, Chen et al. [28] also combined the network-constrained penalty term with a SVM model and proposed the netSVM method for the purpose of classification. More recently, Sokolov et al. [29] generalized the elastic net penalty term to incorporate pathway knowledge and then combined the proposed penalty term with an objective function to select relevant genes. The proposed term is referred to as the generalized elastic net (gelnet) function, and it includes the elastic net as a special case. The big disadvantage of a penalty method is that its computing burden is moderate or even heavy. Three separate figures (Figures 2–4) were made to elucidate these three major types of pathway-guided gene selection methods in detail. A review of typical examples in each category is given in Table 1.

### 3.5. Penalty Function

Given the fact that penalization plays a critical role in both the pathway-guided gene selection and in bilevel selection methods, we discuss the commonly used penalty terms in both methods in the following sections.

#### 3.5.1. Network-Constrained Penalty

For a penalty method, one well-known network-constrained penalty term was proposed by [27]. It is notated as

(1) $$\begin{array}{c} \underset{u\sim v}{\sum }{\left(\;\frac{\beta_{u}}{\sqrt{d_{u}}}-\frac{\beta_{v}}{\sqrt{d_{v}}}\;\right)}^{2}w\left(\;u,v\;\right) \end{array}$$

Here w(u,v) denotes the weight of edge (u, v). It usually takes the value of 1 if gene u and gene v are connected, 0 otherwise. The degree of gene u (denoted as d_u_) is the sum of edge weights over all vertices connected with u, i.e., ∑_*u*~*v*_*w*(*u*, *v*). This term introduces a smooth solution of *β* coefficients (which represent the association magnitudes and directions of genes with the outcome) on the network via penalizing the weighted sum of squares of the scaled difference of the coefficients between connected genes. Li & Li [27] specifically stated that scaling the *β* coefficients using their respective degrees of freedom on the network “allows the genes with more connections to have larger coefficients so that small changes of expressions of such genes can lead to large changes in the response”. Several studies had adopted and imposed this penalty term on different objective functions. For instance, Chen et al. [28] had imposed this constraint on a support vector machine (SVM) model and developed a new approach called the network-constrained support vector machine (netSVM) method. For a more detailed description on the penalty functions at the pathway level, the work by Pan et al. [30] and Table 2 are referred.

#### 3.5.2. General Penalty Framework for a Bilevel Selection Method

For a bilevel selection process, Breheny & Huang [31] presented a general framework of the penalty functions used, which is

(2) $$\begin{array}{c} f_{o}\left(\;\overset{K_{j}}{\underset{k=1}{\sum }}f_{I}\left(\;\left|\;\beta_{j,k}\;\right|\;\right)\;\right) \end{array}$$

where the subscript j, k represents gene k (k=1,2,… K_j_, where K_j_ is the size of gene set j) inside group j (j=1,2,…J, where J is the number of gene sets under consideration). In this formula, an outer penalty function f_o_, e.g., the bridge penalty, is applied to a sum of inner penalties f_I_, e.g., the LASSO. The outer penalty regularizes the coefficients of all genes within the specific get sets while the inner penalty function penalizes on the coefficients before individual genes.  Table 3 summarizes those penalty terms commonly used in a simultaneous bilevel selection process.

**Figure 5:**
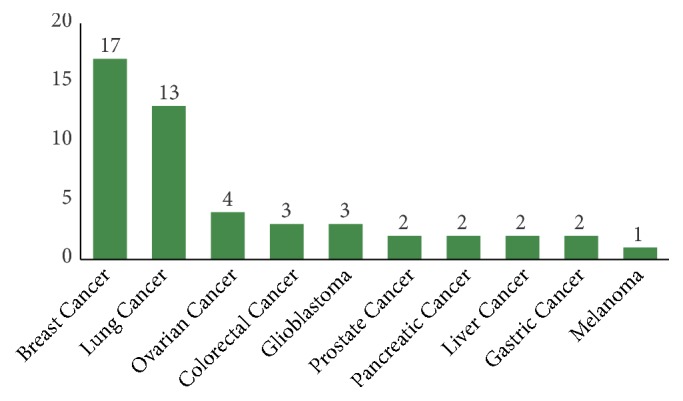
*Statistics for pathway-guided gene selection methods in cancer studies*. A literature search was conducted in the PubMed using keywords of feature selection, gene expression, pathway/network, and cancer. The number of relevant articles stratified by the cancer types under study is given on the top of those bars.

After searching in the literature, it is found that pathway-guided gene selection methods have been widely applied in cancer studies. Specifically, a pathway-guided gene selection algorithm may cast some insight on identifying diagnostic gene signatures capable of distinguishing cancer patients from normal controls; different subtypes of a specific cancer; or histologic stages, or identifying prognostic signatures that predict the survival time of cancer patients. By searching in the PubMed using the keywords of feature selection, pathway/network, gene expression, and cancer and then inspecting their relevance, we found roughly 40 articles which utilize pathway-guided gene selection algorithms to study a variety of cancers.  Figure 5 provides the statistics of these articles by stratifying them according to the cancer types under study. From this figure, it is observed that the most frequently studied cancer types are breast cancer, e.g., the studies by [23, 26, 28, 29, 32–35] and lung cancer, e.g., the studies by [16, 20, 33, 36–40].

Among these studies, the penalty method is the most prevalent method, being followed by the stepwise forward method. This observation provides evidence to support our statement that the strategy of a penalized regression model to select relevant genes has gained increasing attention and the weighting methods have been underutilized compared to the other two categories. Given there are several public repositories such as The Cancer Genome of Atlas (TCGA: https://portal.gdc.cancer.gov/), the Gene Expression Omnibus (GEO: https://www.ncbi.nlm.nih.gov/), and Array Express [41], we believe more investigation will boom to utilize pathway-guided gene selection methods to study other cancer types and other complex diseases.

## 4. Pathway Information 

### 4.1. Topology or Grouping Information

As we mentioned in the early section, different algorithms may account for different pathway knowledge. For examples, some algorithms consider pathway topology information (e.g., which genes are connected to which genes) whereas some ignore it. In the methods that omit topology information, genes are grouped into many gene sets and only the group membership of genes is considered. From the perspective of weighting, the methods using grouping information weigh every gene inside a specific pathway equally while the first type of methods may prioritize the genes with high connectivity level. Based on whether topology information is considered, a pathway-guided gene selection method can be divided into either a functional score based method or a topology-based method. In a functional score based method such as [10, 18], only the grouping membership of genes is considered to generate an evaluation score, with an implicit assumption that all genes inside a specific pathway coregulate/cofunction together. In contrast, in a topology-based method such as [28] more structured pathway knowledge rather than grouping information is considered.

### 4.2. Data-Driven versus Canonical Pathways

Several studies, e.g., [42, 43], have concluded that pathway-guided gene selection does not outperform classic gene-based feature selection methods in terms of predictive accuracy. This inferiority may be explained by the fact that the pathway knowledge retrieved from those canonical pathway databases/knowledge-bases such as the Kyoto Encyclopedia of Gene and Genomes (KEGG) [44], Gene Ontology (GO) [45], and Reactome [46] conveys no or limited meaningful information for a specific dataset or condition/disease. In contrast, the pathways constructed in a “data-driven” way may be more informative for the diseases under investigation and thus be preferred over the canonical pathways. Here, “data-driven” means that specific data of a specific condition/disease are used to build up pathways [47, 48]. The construction for those data-driven pathways is usually accomplished with the aids of a coexpression module detection technique, e.g., the Weighted Gene Coexpression Network Analysis (WGCNA) [49] and Algorithm for the Reconstruction of Accurate Cellular Network (ARACNE) [50]. Also, there exist some elegant algorithms, e.g., [51, 52], that are able to figure out grouping structures and carry out feature selection simultaneously. No matter which strategy it takes, in the “data-driven” pathway construction pathway structure is inferred from data.

On the other hand, data-driven pathways provide no information about causality given they cannot determine genes' positions in the whole network and thus cannot distinguish the regulatory/upstream genes apart from the regulated/downstream genes [61]. In addition, different form the static representations of the biological pathways, say, protein-to-protein interaction, metabolic networks, or signaling networks curated in those canonical databases, these data-driven pathways vary from data to data and thus may be subject to random noises and difficult to be interpretable from a biological point of view [62]. Finally, the resulting models by using data-driven pathways may be subject to overfitting since the build-up of coexpression/coregulation modules and the selection of relevant features are usually carried out on the same dataset.

Therefore, a through evaluation on which pathways are used during data analysis is highly desirable, in order to maximize the information extraction and to infer true biological meaning.

## 5. Potential Research Area

So far, the feature selection algorithms we have talked are mainly for cross-sectional data in which data were collected at a single time point. The number of feature selection algorithms for longitudinal data in which the subjects were followed up across time and the corresponding data were collected at different time points is not comparable to that of cross-selection data. To name several, the EDGE method [63, 64], the Generalized Estimating Equation- (GEE-) based screening procedure by [65], the penalized-GEE method [66], and the Penalized-GEE with Grid Search (PGS) method by [67] are included in this small-sized list of longitudinal feature selection algorithms.

As far as the pathway-based feature selection algorithms are considered, to the best of our knowledge, one of our extensions to the SAMGSR method [10], the two-level SAMGSR method, is the only approach that incorporates pathway information to specifically deal with longitudinal data [68]. In the two-level SAMGSR method, the reduction step of the SAMGSR algorithm [10] is applied twice hierarchically. Specifically, the selected gene sets are further reduced to their respective important components, i.e., genes, and then the important time points in selected genes are identified subsequently. Nevertheless, the two-level SAMGSR only considers the grouping membership information. The results of several real-world applications where the diseases under investigation include non-small-cell lung cancer, multiple sclerosis, and traumatic injury [36, 68, 69] have suggested the performance improvement for a pathway-guided method only considering the grouping information over a conventional method may be trivial. In contrast, when a pathway-based method accounts for extra pathway knowledge such as the connectivity information among genes and regulation direction recording which genes regulate which genes, its performance might be promoted dramatically.

One major finding of our previous studies [68, 70] is that the gene expression profiles across different time points may be regarded as a gene set and then some suitable pathway analysis methods may be adopted to select relevant genes for longitudinal data. In the light of this, summary scores at the pathway level such as means, medians [71], the first principal components (PCs) [72], and the sign averages [17, 73] which average out the signed expression values, with signs indicating the association directions between genes and outcome, or more statistically complicated ones like the pathway deregulation scores (PDS) [74], may be chosen to generate pseudo genes as representatives for respective pathways, and then a longitudinal feature selection process has been downgraded to a classic feature selection process.

Furthermore, one may be also interested in finding those monotonically changed genes as the disease progresses, which may be regarded as a special case of the feature selection for longitudinal data. The word “monotonic” means descending or ascending change patterns across time or stages/grades. To the best of our knowledge, no pathway-based algorithms have been proposed to tackle this specific topic. Therefore, more investigation is warranted to explore if a pathway-guided method is superior to a conventional method such as [75] in selecting monotonic genes. In summary, pathway-guided gene selection may play more roles on identifying potential biomarkers for longitudinal omics data.

## 6. Conclusions 

In this article, we present a review on pathway-based feature selection algorithms. First, based on to what a feature corresponds, pathway-based feature selection methods are classified into three categories, pathway-level selection methods, bilevel selection methods, and pathway-guided gene selection methods. By focusing on the selection of individual genes where pathway information is incorporated as a prior to guide feature selection, pathway-guided gene selection methods were reviewed and discussed in detail. Additionally, given the importance of penalization in the process of feature selection, the commonly used penalty functions in a pathway-guided gene selection method were reviewed. Last, we point out one potential research area in which pathway-guided gene selection deserves more attention, namely, longitudinal gene expression data analysis.

We believe this review provides valuable insights for computational biologists/biostatisticians and stimulates them to develop more elegant pathway-guided gene selection algorithms. The development and wide application of such algorithms to reveal underlying pattern, elucidate the etiology and progression of complex diseases, and guide more “personalized” treatment strategies will contribute substantially to make biology more computable.

## Figures and Tables

**Table 1:** A selective review of pathway-guided gene selection algorithms.

Reference	Brief description of the proposed method and its characteristics	Category
Zhu et al. [26]	The proposed network-based SVM method combines the network-constrained penalty (see equation (1)) with a SVM model to carry out feature selection and classification. It makes SVM models capable of carrying out feature selection; the network-constrained penalty gives heavier weights to genes with more direct neighbors (thus increases the chance of such genes being selected) and encourages a grouping effect. But the method only deals with binary classification and considers immediate neighbors.	Penalty
Chen et al. [28]	The netSVM method also combines the network-constrained penalty (see equation (1)) with a SVM model. Its advantages and disadvantages are similar to the network-based SVM method by Zhu et al [26] (see above)	Penalty
Sokolov et al. [29]	The generalized elastic net penalty function is given and combined with an objective function to select important genes. This is named as the GELnet method. The authors claimed that this penalty function includes many well-known penalty terms and the method is so flexible that it can deal with many outcome types. There is an independent R package (i.e., gelnet) to implement this method, but now this package can only conduct binary classification.	Penalty
Zhang et al. [53]	The Net-Cox method adds a network-constrained penalty term to the corresponding partial likelihood function of a Cox model, aiming to select important prognostic genes The Matlab codes are available online, making the implementation of this method easy. This method only considers direct neighbors.	Penalty
Bandyopadhyay et al. [32]	After ranking genes in a pathway according to their marginal classification power, the proposed BPFS method starts from the gene with the largest power and then adds genes The authors claimed that this method goes beyond the immediate neighbors and considers redundant gene elimination. Also, missing genes in the pathway databases are mapped to the network using a probabilistic technique. However, the method is hard to comprehend, and no codes are available.	Stepwise forward
Lee et al. [33]	In each pathway, the method reorders genes according to their t-scores, and then the subset of genes whose combined expression has optimal discriminative power called CORGs is identified. Only the membership of genes is considered. The method is simple and easy to implement.	Stepwise forward
Razi et al. [34]	The proposed NBCG method starts with a seed gene and traverses the network to find the optimal subset on the basis of Shapley value. The method uses the concept of Shapley value to take into account the collective power of the resulting gene subset. The choice of a seed gene may result in excluding a gene subset with subtle individual effects but significant concordant effect.	Stepwise forward
Wu et al. [54]	The shortest path method (with well-known genes related to the disease under study, i.e., gastric cancer as seeds) is used to mine candidate genes and the combination of random forest +incremental feature selection is used to obtain the optimal subset. The proposed method considers topology information of a network. The use of a wrapper method (RF+IFS) and permutation tests may slow the method down.	Stepwise forward^1^
Tian et al. [20]	The weighted-SAMGSR method extends the SAMGSR algorithm by weighing SAMGS statistics according to genes' connectivity levels in the network. The method considers both the membership information and the connectivity level, and can handle two-class and multiple-class classification. The R-codes are available in the supplementary material. Computing time is a big concern since permutation tests are needed to calculate p-values of test statistics.	A hybrid of weighting and stepwise forward
Johannes et al. [23]	The RRFE method uses the GeneRank algorithm to alter the ranking criterion of the SVM-RFE algorithm and selects a subset with the best discriminative power. Weighing the coefficients of SVM models with their GeneRanks to increase the probability of a gene with more connected genes being selected, an independent R package (i.e., pathClass) is provided to implement this method. The method only considers how many direct neighbors a gene has and ignores topology information completely.	Weighting
Chan et al. [39]	The wgSVM-SCAD method weighs the expression values of genes in a pathway according to their t-values and then uses a penalized SVM model (with SCAD penalty) to identify relevant genes. The proposed method only considers membership information and the weights are only based on the relevance score (i.e., t-values) instead of pathway information.	Weighting
Tian et al. [16]	Using sign averages of all genes inside a gene set to represent corresponding gene set, the proposed methods (i.e., one forward bi-level selection method and one backward bi-level selection method) filter out insignificant gene sets and insignificant genes in a specific order. The sign average metric provides a better representation of a gene set than mean, median and the first PC. The proposed methods only consider membership information.	Bi-level selection
Lim and Wong [19]	In both FSNet and PFSNet methods, a fuzzy value is assigned to each gene for each sample and then majority voting is used to determine important genes. The codes are available online. The proposed methods only consider the gene grouping membership information.	Bi-level selection

Note: Bilevel selection algorithms are regarded as a special case of pathway-guided gene selection algorithms.

^1^Can be loosely categorized into the indicated category (e.g., stepwise forward).

**Table 2:** Penalty terms used in the penalty methods.

Methods	Mathematical notation	Characteristics
Li & Li, 2008 [27]	$\underset{u\sim v}{\sum }{\left(\frac{\beta_{u}}{\sqrt{d_{u}}}-\frac{\beta_{v}}{\sqrt{d_{v}}}\right)}^{2}w\left(u,v\right)$ Here, d_u_ is the degree of freedom for gene u, recording the sum of weights for all genes connected to gene u. *w*(*u*, *v*) is the weight for the edge between genes u and v.	Aims at smoothing the *β* coefficients over the network, ignoring that neighboring genes might have *β*'s in opposite directions.

[55]	$\underset{u\sim v}{\sum }{\left(\frac{{sign(\tilde{\beta_{u}})\beta }_{u}}{\sqrt{d_{u}}}-\frac{{sign(\tilde{\beta_{v}})\beta }_{v}}{\sqrt{d_{v}}}\right)}^{2}w\left(u,v\right)$ Here, $\tilde{\beta_{u}}$ is the estimated value of *β* coefficient for gene u, and sign (x) represents the sign of x, if x>0 sign(x)=1; x<0 sign(x)=-1; otherwise sign(x)=0.	Accounts for that two connected genes might have *β*'s with different signs, but may not work well since it is difficult to estimate the signs for *β*'s.

[30]	$\underset{u\sim v}{\sum }{\left[{\left(\frac{\beta_{u}}{\sqrt{d_{u}}}\right)}^{\gamma }+{\left(\frac{\beta_{v}}{\sqrt{d_{v}}}\right)}^{\gamma }\right]}^{1/\gamma }w\left(u,v\right),\;\;and\;\;\gamma >1$	Shrinks the weighted *β*'s of two neighboring genes towards each other, but the estimates may be severely biased.
[26, 56]	for *γ* = *∞*, it becomes $\underset{u\sim v}{\sum }\max\left(\frac{{|\beta }_{u}|}{\sqrt{d_{u}}},\frac{{|\beta }_{v}|}{\sqrt{d_{v}}}\right)w\left(u,v\right)$	A 2-step procedure is used to reduce biases; it is proved that this performs better than that with smaller *γ*

[57]	$\underset{u\sim v}{\sum }\left|I\left(\frac{\left|\beta_{u}\right|}{d_{u}}\neq 0\right)-I\left(\frac{\left|\beta_{v}\right|}{d_{v}}\neq 0\right)\right|w\left(u,v\right)$ Here, I (x) is an indicator. If the condition x is true I(x)=1, otherwise its value is 0.	Encourages simultaneous selection of neighboring genes in the network. But the Indictor function I is not continuous and thus needs special care.

The generalize elastic net: [29]	$\lambda_{1}\underset{u}{\sum }D_{u}\left|\beta_{u}\right|+\frac{\lambda_{2}}{2}\beta^{T}P\beta$ Here D and P are additional penalty weights for individual genes (gene-level penalty) and gene pairs (pathway-level penalty).	Includes the network-constrained penalty term by [27] as a special case, capable of accommodating any positive semi-definite measure of dissimilarity between pairs of genes.

**Table 3:** Penalty terms used in the bilevel selection methods.

Methods	Mathematical notation	Characteristics
Group LASSO [58]	General form*f*_*o*_(∑_*k*=1_^*K*_*j*_^*f*_*I*_(|*β*_*j*,*k*_|)) See equation (2) for what f_o_, f_I_ and *β*_j,k_ represent Outer bridge penalty + inner ridge penalty	It cannot identify the important genes within the selected gene sets and thus is actually incapable of bilevel selection and also heavily shrinks large coefficients (leading to estimate biases for large coefficients)
Group bridge [59]	Outer bridge penalty+ inner LASSO penalty	It can provide sparse solutions at both pathway and gene levels, but it is associated with big empirical difficulties since the bridge penalty is not everywhere differentiable.
Group MCP [31]	Outer MCP penalty+ inner MCP penalty	Allow coefficients to grow large and groups to remain sparse.
Group exponential LASSO [18]	Outer exponential penalty + inner LASSO penalty	A decay parameter controls the degree to which gene selection is coupled together within gene sets and has several advantages over the other composite penalty term such as group bridge.
Sparse group LASSO [60]	$\lambda_{1}\underset{j}{\sum }\underset{k}{\sum }\left|\beta_{j,k}\right|+\lambda_{2}\overset{J}{\underset{j=1}{\sum }}{\left\|\beta_{j}\right\|}_{2}$ taking the additive format	Convex and thus highly likely to get the global minimum, but extra care is needed since the group coordinate descent algorithms cannot be applied.

Note: the general formatting for group LASSO, group bridge, and group MCP was given by Breheny & Huang [31]. It is too general to guarantee all combinations of outer and inner penalties produce sensible models. Thus the second general form was proposed by Huang et al. [59] to address this issue specifically.
